# On the Rupture of Aneurisms in the Brain

**Published:** 1826-07

**Authors:** 


### RUPTURE OF ANEURISMS IN THE BRAIN.—M. SERRES.

M. Serres has divided apoplexies, accompanied by extravasation, into two kinds, hemato-meningeal, and hemato-encephalic. The former may be the consequence of rupture of a vein, an artery, or an aneurismal tumour. The *latter* may have its seat in the cerebellum, also, and then our author denominates it hemato-cerebellic, and so on. These two kinds of extravasation, he observes, differ essentially according to their seats; that is, according as they are in the meninges or the medullary structure. They differ, he avers, in their symptoms. In the former, or hemato-meningeal extravasation, there is no paralysis of the voluntary muscles. In the hemato-encephalic species, there is always more or less paralysis. In both cases, the blood effused is in a clot; but in the latter kind (hemato-encephalic) it is lodged in an excavation, more or less profound, in the encephalon. In the former (hemato-meningeal) the blood is extended over the surface of the brain, and in the interior of the ventricles, between the tunica arachnoidea and the pia mater, penetrating wherever these membranes penetrate. These last cases are rare, and those cases dependent on rupture of an aneurismal artery are rarer still. The following cases are, therefore, interesting, and are deserving of record.

*Case* 1. (Hemato-meningeal.) G. Espert, 59 years of age, of very robust constitution, had long been subject to a sense of weight in the head, augmented by any violent exercise or excess in eating or drinking. On the 4th February, he became affected with pneumonia, for which he was received into La Pitié, and was cured by bleeding and proper remedies. On the 26th, when he was ready to quit the hospital, he received the news of a domestic affliction, which caused a violent mental emotion, and fainting that continued for some time. M. Serres found him in the following condition the next day:—face flushed—jugulars turgid—breathing high—pulse hard, full, and quick—stupid appearance.—A copious bleeding relieved these symptoms. 28th, Complete apoplexy—the automatic movements, when excited, were very feeble—redness and tumefaction of the face. Died suddenly this day.

*Dissection.* Before a class of students M. Serres prognosticated that the disease was meningeal apoplexy. On removing the skull, a large quantity of blood was found at the basis cranii, coagulated into laminated clots. On removing the brain, it was discovered that there was an aneurism of the basilary artery, an inch in diameter, and the pouch equal to a pullet's egg. On one side, there was an opening, and through this full a pound of blood had escaped, following the meninges, and penetrating into the ventricles which were completely distended.

*Case* 2. This was a female, 59 years of age, who fell down senseless, on the 3d January, 1826, and was brought to La Pitié the next day, where the house-surgeon applied leeches to the head. 5th, M. Serres found the patient in a state of stupor—face flushed—pulse small and not quick. There were doubts whether any paralysis existed, and, therefore, our author hesitated to give his prognosis as to the seat of the apoplectic cause.

*Dissection.* When the skull-cap was removed, the meningeal veins were observed to be exceedingly turgid, and a sanguineous extravasation was spread, like a sheet, over the hemispheres, dipping down into the anfractuosities. The blood was situated between the pia mater and tunica arachnoidea. By carefully removing the brain and examining the arteries, there was found an aneurism of one of the vessels forming the circulus Willisii, which had burst and produced this meningeal apoplexy. The blood had filled the ventricles, and was prodigious in quantity.

The two cases, above described, are curious specimens of pathology, since rupture of an aneurismal artery is seldom likely to produce this kind of extravasation between the meninges, appearing like a sheet of blood, and puzzling (as we have more than once seen) some good anatomists in this country.—*Archives, Mars*, 1826.

---

